# Concordance Analysis Between Sputum and Bronchoscopic Specimens on Nontuberculous Mycobacteria Pulmonary Disease

**DOI:** 10.3390/jcm15010296

**Published:** 2025-12-30

**Authors:** Sojung Park, Jin Hwa Lee, Nam Eun Kim, Yune-Young Shin

**Affiliations:** Division of Pulmonary and Critical Care Medicine, Department of Internal Medicine, College of Medicine, Ewha Womans University, Seoul 07985, Republic of Korea; spark85@ewha.ac.kr (S.P.); jinhwalee@ewha.ac.kr (J.H.L.);

**Keywords:** diagnosis, nontuberculous mycobacteria, *Mycobacterium avium* complex, sputum, bronchial washing, prognosis

## Abstract

**Background/Objectives**: This study evaluated the concordance between sputum and bronchoscopic specimens in diagnosing nontuberculous mycobacteria (NTM) pulmonary disease. **Methods**: We retrospectively analyzed patients with NTM isolated from respiratory specimens between 2010 and 2022. Our analysis assessed species concordance across the two diagnostic methods and compared clinical outcomes between patients with multiple positive cultures and those with a single positive culture. **Results**: A total of 400 patients were included, 100 of whom underwent bronchoscopy. Among these, 61 demonstrated concordant NTM species between sputum and bronchoscopic specimens, while 38 had NTM cultured from only one source. One patient showed a discordant result, with *Mycobacterium abscessus* isolated from bronchoalveolar lavage and *Mycobacterium avium* from sputum. Multivariate analysis identified several factors associated with radiologic progression or the need for treatment: body mass index (HR, 0.847; 95% CI, 0.794–0.902; *p* < 0.001), membership in the single-isolation group (HR, 0.400; 95% CI, 0.184–0.871; *p* = 0.021), and fibrocavitary radiologic type (HR, 2.318; 95% CI, 1.470–3.655; *p* < 0.001). **Conclusions**: Only a small number of cases showed different NTM species identified by sputum and bronchoscopy.

## 1. Introduction

The prevalence of nontuberculous mycobacterial (NTM) pulmonary disease (PD) is rising worldwide [1,2,3,4]. As recognition of NTM as significant pathogens grows, accurate diagnosis and effective treatment of NTM-PD have become critical medical priorities. Diagnosis of NTM-PD requires fulfillment of clinical, microbiological, and radiological diagnostic criteria [5,6,7]. In particular, microbiological confirmation demands positive results from at least two separate sputum or one bronchial wash or lavage specimen.

A single isolation of NTM from expectorated sputum may be insufficient for diagnosis, as respiratory specimens are prone to environmental contamination. Accordingly, current guidelines recommend bronchoscopy for patients suspected of NTM-PD who either fail to produce culture-positive sputum or are unable to expectorate sputum [6,7]. Nevertheless, the growing prevalence of NTM infections and advances in specimen processing suggest that even a single positive result may be clinically significant. Sin et al. reported that NTM-PD diagnosed using methods other than sputum culture was associated with slower disease progression and a slower risk of advancement compared with NTM-PD diagnosed through sputum culture [8].

This study compared the diagnostic agreement on NTM species between sputum and bronchoscopic specimens in patients with suspected NTM-PD. We also examined radiologic progression and treatment requirements in patients diagnosed using two or more respiratory specimens versus those diagnosed with a single specimen.

## 2. Materials and Methods

### 2.1. Study Design and Population

We conducted a retrospective investigation of patients at Ewha Womans University Mokdong Hospital in Seoul, South Korea, between January 2010 and December 2022, in whom NTM were isolated from sputum, bronchial wash, or lavage specimens. We excluded patients without radiological findings suggestive of NTM-PD, those with positive results only on the NTM PCR assay, those with a history of NTM-PD treatment before the study period, patients diagnosed with pulmonary tuberculosis (TB) within one year before or after NTM isolation, and those with isolation of *Mycobacterium gordonae*. We analyzed the results of all bronchoscopies performed in the included patients, regardless of the clinical indication for the procedure.

We compared NTM species isolated from sputum and bronchoscopic specimens. Patients were classified into a single-isolation group or a multiple-isolation group depending on whether NTM was identified only once (including bronchoscopic specimens) or on multiple occasions. We then evaluated differences in baseline demographics and clinical outcomes between the two groups.

The Institutional Review Board of the Ewha Womans University Mokdong Hospital approved the study and waived the informed consent requirement due to its retrospective design (EUMC 2025-10-041).

### 2.2. Data Collection

We retrospectively reviewed baseline demographics, including age, sex, body mass index (BMI), smoking status, and underlying lung disease, and compared them between the two groups. All NTM species isolated during follow-up were investigated. To assess concordance, we included NTM species identified from sputum and from bronchial wash or lavage specimens collected within a one-year interval. Sputum AFB culturing was performed multiple times in most patients, but only once in cases where patients could not expectorate sufficient sputum. Chest computed tomography (CT) scans obtained during follow-up were also collected. Radiographic features, including radiographic disease type, extent, and the presence of cavities or bronchiectasis, were evaluated at baseline and during follow-up. The data cutoff date was 31 December 2024.

### 2.3. Statistical Analysis

We compared categorical variables between the two groups using the Pearson’s chi-square test or Fisher’s exact test, and continuous variables using Student’s *t*-test or the Mann–Whitney U test. Crude incidence rates were calculated as the number of deterioration events per person-year of follow-up. Differences between groups were assessed using Poisson-based incidence rate ratios with 95% confidence intervals (CIs). The Kaplan–Meier method was used to compare the time to radiologic deterioration or the need for treatment between the two groups, and the log-rank test was applied for statistical comparison. Significant variables in the univariate analyses (*p* < 0.1) were evaluated in a multivariate analysis using the Cox proportional hazard model. The results were expressed as hazard ratios (HRs) with 95% CIs. All significance tests were two-sided, and differences were considered statistically significant at *p* < 0.05. Statistical analyses were performed with SPSS software, version 27.0 (SPSS Inc., Chicago, IL, USA).

## 3. Results

### 3.1. Baseline Characteristics

We included 400 patients in the present study. Of these, 97 were classified into a single-isolation group, and 303 into a multiple-isolation group. Baseline characteristics are summarized in Table 1. The mean age of the 400 patients was 65.6 ± 13.0 years, and 255 patients (63.7%) were women. Baseline characteristics did not differ significantly between the two groups, except for BMI, which was lower in the multiple-isolation group (20.5 ± 3.3 vs. 21.5 ± 3.4 kg/m^2^, *p* = 0.009). The prevalence of bronchiectasis was also significantly higher in the multiple-isolation group (244/303, 80.5%) compared with the single-isolation group (66/97, 68.0%, *p* = 0.010). In addition, acid-fast bacilli smear positivity occurred more frequently in the multiple-isolation group (100/303, 33.0%) than in the single-isolation group (7/97, 7.2%, *p* < 0.001).

### 3.2. Comparison of Microbiological Findings

A total of 100 patients underwent bronchoscopy, and 85 (85/100, 85.0%) had NTM strains isolated from bronchial wash or lavage. The most common reason for performing bronchoscopy was differential diagnosis of TB (38/100, 38.0%), followed by evaluation of endobronchial lesion for hemoptysis (32/100, 32.0%) and microbiological diagnosis of NTM-PD (18/100, 18.0%). The distribution of NTM species cultured from all respiratory and bronchoscopic specimens is shown in Table 2. Concordant NTM species between sputum and bronchoscopic specimens were observed in 61 patients. Only one patient demonstrated a discordant result, with *Mycobacterium abscessus* isolated from bronchoalveolar lavage and *Mycobacterium avium* from sputum. NTM was detected exclusively in bronchoscopic specimens in 23 patients. Among the 15 patients with NTM detected only in sputum, 12 had the same species isolated more than once, while three had a single isolation. Multiple NTM species were identified in respiratory specimens from 33 patients (33/400, 8.3%; Supplementary Table S1). Of the nine patients with multiple NTM species in sputum cultures and positive bronchoscopic cultures, five showed concordant multiple NTM species in both specimen type, whereas the remaining four had only one of the sputum-isolated species detected bronchoscopically. During follow-up, 36 patients (36/400, 9.0%) had a different NTM species isolated over a one-year interval.

### 3.3. Radiologic Progression

**Figure 1:**
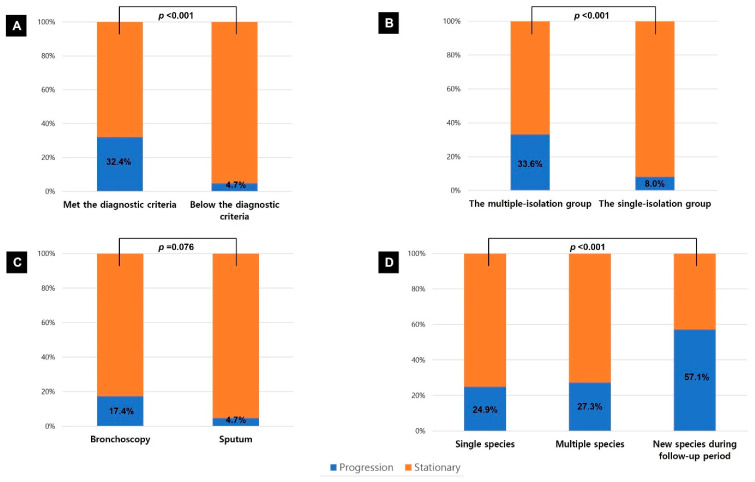
Radiologic progression or treatment requirement in patients with nontuberculous mycobacterial pulmonary disease, shown according to the American Thoracic Society/Infectious Diseases Society of America statement criteria (**A**), multiple vs. single-isolation group (**B**), specimen type within the single-isolation group (**C**), and species diversity (**D**).

**Figure 2:**
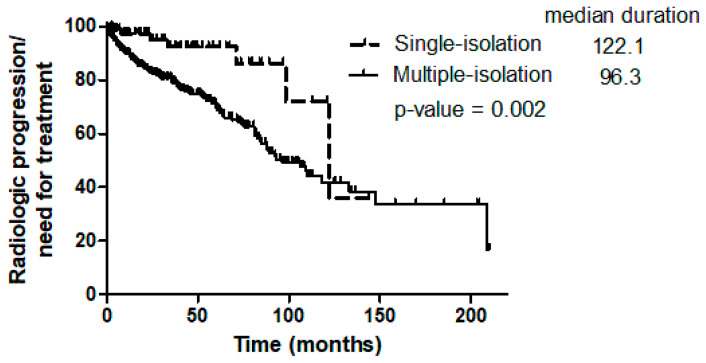
Time from first nontuberculous mycobacterium isolation to radiologic deterioration or treatment initiation in patients with nontuberculous mycobacterial pulmonary disease.

Chest CT was performed in 382 patients during follow-up to assess radiologic status. No deterioration was observed in the patient whose bronchoscopic and sputum results were discordant. Patients who met the American Thoracic Society/Infectious Diseases Society of America statement criteria (103/318, 32.4% vs. 3/64, 4.7%, *p* < 0.001; Figure 1A) and those in the multiple-isolation group (99/295, 33.6% vs. 7/87, 8.0%, *p* < 0.001; Figure 1B) showed significantly higher rates of radiologic deterioration or required treatment compared with patients who did not meet the criteria or belonged to the single-isolation group. The crude incidence rate of radiologic deterioration or the need for treatment was significantly higher in the multiple-isolation group than in the single-isolation group (0.0839 vs. 0.0272 events per person-year; incidence rate ratio, 3.09 [95% CI, 1.43–6.65; *p* = 0.004]; Table 3). The median time from diagnosis to radiologic deterioration or treatment initiation was also shorter in the multiple-isolation group compared with the single-isolation group (96.3 vs. 122.1 months; *p* = 0.002; Figure 2).

Bronchoscopy itself was not significantly associated with prognosis. Within the single-isolation group, patient with NTM isolated from bronchoscopic specimens tended to have higher rates of radiologic deterioration or treatment requirement than those with NTM isolated from sputum, but the difference was not statistically significant (4/23, 17.4% vs. 3/64, 4.7%, *p* = 0.076; Figure 1C). Patients in whom a different species was isolated over a one-year interval showed a higher rate of radiologic deterioration (16/28, 57.1%) compared with those with persistent isolation of a single species (80/321, 24.9%) or multiple species from baseline (9/33, 27.3%, *p* = 0.001; Figure 1D).

Fibrocavitary features on chest CT were more frequent in the multiple-isolation group than in the single-isolation group, both in the overall cohort (52/303, 17.2% vs. 8/97, 8.2%, *p* = 0.016) and in patients with *Mycobacterium avium* complex (MAC) (36/236, 15.3% vs. 6/75, 8.0%, *p* = 0.033; Table 4). In multivariate analysis, BMI (HR, 0.847; 95% CI, 0.794–0.902; *p* < 0.001), membership in the single-isolation group (HR, 0.400; 95% CI, 0.184–0.871; *p* = 0.021), and fibrocavitary type (HR, 2.318; 95% CI, 1.470–3.655; *p* < 0.001) were significantly associated with radiologic progression or the need for treatment (Table 5).

## 4. Discussion

In the present study, identical NTM species were cultured from both bronchoscopic and sputum samples in all but one patient. To our knowledge, this is the first investigation to evaluate the concordance between NTM species isolated from bronchoscopic specimens and those isolated from sputum. The study aimed to determine whether a single positive sputum culture in patients with radiologic findings suggestive of NTM-PD could represent a true pathogen rather than environmental contaminant.

NTM are ubiquitous in the environment and not always pathogenic. Current diagnostic criteria require multiple positive sputum cultures, yet clinical evidence supporting this requirement remains limited–even in patients with radiologic findings consistent with NTM-PD [5,6]. A study on the natural history of MAC-PD reported that the stationary group had less frequent NTM isolation, higher BMI, and a non-fibrocavitary type compared with the progressive group [9]. Furthermore, both our study and previous reports found no significant difference in prognosis between patients with a single positive sputum culture and those with a single positive bronchoscopy culture [8,10]. Meanwhile, it has been reported that 10–15% of patients have multiple NTM species isolated [11,12,13]. In our study, even among patients with multiple species, concordance between sputum and bronchoscopy remained high, and 90.9% (30/33) met diagnostic criteria by having positive cultures on more than two occasions. Given this high concordance, bronchoscopy may not be necessary in patients with a single positive sputum culture, compatible radiologic findings, and a stable clinical course–even when confirming diagnosis or evaluating the possibility of multiple NTM species.

Bronchoscopy remains a relatively safe diagnostic procedure for identifying NTM species [14,15,16,17,18]. One study reported a 95% diagnostic yield from bronchoalveolar lavage specimens, but most studies have shown detection rates ranging from 38% to 57% [16,17,18,19]. Because many patients with NTM-PD have underlying lung diseases such as bronchiectasis, improper selection of sampling sites may increase the risk of false-negative results. Gu et al. demonstrated that additional post-bronchoscopy sputum examination increased diagnostic yield by 3.5%, while Holt et al. reported detection rates exceeding 80% with serial sputum induction [16,20].

Low BMI has been linked to poor outcomes, including radiologic progression, treatment failure, and in–hospital mortality [8,9,21,22]. Several studies have also shown that low BMI is associated with higher mortality in patients with other chronic lung diseases, such as TB, chronic obstructive pulmonary disease and interstitial lung disease [23,24,25,26]. Poor nutritional status may compromise immune function, facilitating the progression of NTM-PD, while an increased NTM burden may manifest as weight loss among systemic symptoms. Patients in the multiple-isolation group demonstrated higher rates of bronchiectasis and a tendency toward more frequent bronchial artery embolization, suggesting more severe chronic airway inflammation, increased sputum production, and a higher rate of acid-fast bacilli smear positivity, suggesting a greater NTM burden. Although direct evidence linking chronic airway inflammation or high NTM burden to reduced BMI is lacking, a nationwide study reported that BMI tends to recover after anti-TB treatment in patients with TB [23]. Thus, low BMI may serve as a potential indicator of disease progression in NTM-PD. Further studies are needed to determine whether nutritional support improves clinical outcomes in these patients.

Our study has several limitations. First, as a retrospective analysis, some patients were not followed for sufficient periods, and chest CT scans were not performed at regular intervals. Data on respiratory symptom severity and reasons for initiating treatment were also incomplete. Although the shorter follow-up duration in the single-isolation group may reflect milder symptoms, this difference could have influenced the comparison of clinical outcomes. Second, bronchoscopy was performed in only a subset of patients, and the relatively small sample size raises the possibility of selection bias. As some bronchoscopies were performed for reasons other than diagnosing NTM-PD, the diagnostic yield of bronchoscopy may have been underestimated. Because this study relied solely on retrospective medical record review, further larger studies are needed to statistically validate concordance between the two diagnostic methods. Third, before 2018, our institution could not reliably differentiate *Mycobacterium abscessus* from *Mycobacterium massiliense* due to limitations in the laboratory. As these subspecies have distinct microbiologic characteristics and clinical outcomes, this limitation may have affected the analysis of prognostic factors.

In conclusion, only a few cases showed different NTM species between sputum and bronchoscopic specimens. With the rising prevalence of NTM infections and advances in specimen processing, even a single positive culture may represent clinically meaningful NTM-PD with a favorable prognosis.

## Figures and Tables

**Table 1:** Baseline characteristics of patients with nontuberculous mycobacterial pulmonary disease.

	Total	Multiple-Isolation	Single-Isolation	*p*-Value
	n = 400	n = 303	n = 97	
Age, years	65.6 ± 13.0	66.1 ± 13.0	64.1 ± 13.1	0.192
Sex, women	255 (63.7)	193 (63.7)	62 (63.9)	0.969
BMI, kg/m^2^	20.8 ± 3.4	20.5 ± 3.3	21.5 ± 3.4	0.009
Smoking status (n = 397)	397	301	96	
Current smoker	24 (6.0)	18 (6.0)	6 (6.3)	
Ex-smoker	80 (20.2)	66 (21.9)	14 (14.6)	
Never smoker	293 (73.8)	217 (72.1)	76 (79.2)	
Smoking, pack-years	7.5 ± 16.0	8.2 ± 16.7	5.9 ± 13.6	0.173
Underlying diseases				
Bronchiectasis	310 (77.5)	244 (80.5)	66 (68.0)	0.010
History of tuberculosis	87 (21.8)	71 (23.4)	16 (16.5)	0.149
COPD	65 (16.3)	47 (15.5)	18 (18.6)	0.479
Interstitial lung disease	14 (3.5)	11 (3.6)	3 (3.1)	0.802
Lung cancer	4 (1.0)	3 (1.0)	1 (1.0)	0.972
Hemoptysis	103 (25.8)	83 (27.4)	20 (20.6)	0.184
History of BAE	35 (8.8)	31 (10.2)	4 (4.1)	0.064
Lymphocyte *, 10^3^/uL	1.75 ± 0.68	1.68 ± 0.63	1.95 ± 0.77	<0.001
Immunocompromised status ^†^	11 (2.8)	6 (2.0)	5 (5.2)	0.145
No. of AFB smear/culture ^‡^	5.2 ± 3.7	5.9 ± 3.8	2.9 ± 2.2	<0.001
No. of AFB smear/culture, BFS ^§^	0.3 ± 0.4	0.2 ± 0.4	0.3 ± 0.4	0.688
AFB smear positivity	107 (26.8)	100 (33.0)	7 (7.2)	<0.001

Data are shown as n (%) per group or means ± standard deviation. * The lymphocyte count at the time of the initial nontuberculous mycobacteria isolation. ^†^ Includes patients receiving immunosuppressants, corticosteroids, or chemotherapy, as well as those with leukemia or human immunodeficiency virus infection. ^‡^ All acid-fast bacilli smear and culture tests performed on respiratory specimens were included, regardless of culture positivity or testing method. For patients who received treatment, only the tests performed before treatment initiation were counted. ^§^ All acid-fast bacilli smear and culture tests performed on bronchoscopic specimens were counted regardless of culture positivity. BMI, body mass index; COPD, chronic obstructive pulmonary disease; BAE, bronchial artery embolization; No., number; AFB, acid–fast bacilli; BFS, bronchoscopy.

**Table 2:** Nontuberculous mycobacteria species isolated from the initial respiratory specimen.

	Total	Multiple-Isolation	Single-Isolation
	n = 400	n = 303	n = 97
All patients			
*Mycobacterium avium*	146 (36.5)	102 (33.7)	44 (45.4)
*Mycobacterium intracellulare*	135 (33.8)	107 (35.3)	28 (28.9)
*Mycobacterium abscessus* complex	52 (13.0)	40 (13.2)	12 (12.4)
*Mycobacterium kansasii*	19 (4.8)	16 (5.3)	3 (3.1)
Miscellaneous	15 (3.8)	8 (2.6)	7 (7.2)
Multiple species	33 (8.3)	30 (9.9)	3 (3.1)
Isolated from bronchoscopic specimens ± sputum	85	62	23
*Mycobacterium avium*	34 (40.0)	21 (33.9)	13 (56.5)
*Mycobacterium intracellulare*	26 (30.6)	20 (32.3)	6 (26.1)
*Mycobacterium abscessus* complex	11 (12.9)	8 (12.9)	3 (13.0)
*Mycobacterium kansasii*	3 (3.5)	3 (4.8)	0
Miscellaneous	1 (1.2)	1 (1.6)	0
Multiple species	10 (11.8)	9 (14.5)	1 (4.3)

**Table 3:** Crude incidence of radiologic progression or treatment requirement and follow-up duration in patients with nontuberculous mycobacterial pulmonary disease.

	Multiple-Isolation	Single-Isolation	*p*-Value
	n = 303	n = 97	
Radiologic deterioration or the need for treatment (n = 382)	99/295 (33.6)	7/87 (8.0)	<0.001
Crude incidence density of deterioration, per PY (n = 382)	0.0839	0.0272	0.004
Follow-up time, months	59.0 ± 47.6	35.2 ± 36.3	<0.001
Lost to follow-up or transferred out *	97 (32.0)	55 (56.7)	<0.001
Death *^,†^	28 (9.2)	10 (10.3)	0.691

Data are shown as n (%) per group or means ± standard deviation. * Includes patients who were lost to follow-up, transferred to another hospital, or died before radiologic deterioration or initiation of treatment. ^†^ Includes all-cause mortality, not limited to deaths attributable to nontuberculous mycobacterial pulmonary disease. PY, person-year.

**Table 4:** Radiologic features of patients with nontuberculous mycobacterial pulmonary disease.

	Total	Multiple-Isolation	Single-Isolation	*p*-Value
Overall patients	400	303	97	0.016
Nodular bronchiectatic type	258 (64.5)	197 (65.0)	61 (62.9)	
Fibrocavitary type	60 (15.0)	52 (17.2)	8 (8.2)	
Unclassifiable	82 (20.5)	54 (17.8)	28 (28.9)	
*Mycobacterium avium* complex	311	236	75	0.033
Nodular bronchiectatic type	200 (64.3)	155 (65.7)	45 (60.0)	
Fibrocavitary type	42 (13.5)	36 (15.3)	6 (8.0)	
Unclassifiable	69 (22.2)	45 (19.1)	24 (32.0)	
*Mycobacterium abscessus* complex	60	48	12	0.470
Nodular bronchiectatic type	43 (71.7)	34 (70.8)	9 (75.0)	
Fibrocavitary type	11 (18.3)	10 (20.8)	1 (8.3)	
Unclassifiable	6 (10.0)	4 (8.3)	2 (16.7)	
*M* *ycobacterium kansasii*	22	18	4	0.354
Nodular bronchiectatic type	9 (40.9)	8 (44.4)	1 (25.0)	
Fibrocavitary type	8 (36.4)	7 (38.9)	1 (25.0)	
Unclassifiable	5 (22.7)	3 (16.7)	2 (50.0)	

Data are shown as n (%) per group or means ± standard deviation.

**Table 5:** Prognostic factors for radiologic progression or treatment needs in patients with nontuberculous mycobacterial pulmonary disease.

	Univariate Analysis		Multivariate Analysis	
	HR (95% CI)	*p*-Value	HR (95% CI)	*p*-Value
Women	0.797 (0.539–1.178)	0.255		
Age, years	1.003 (0.987–1.020)	0.715		
BMI, kg/m^2^	0.832 (0.782–0.884)	<0.001	0.847 (0.794–0.902)	<0.001
Never smoker	0.941 (0.613–1.446)	0.782		
Presence of bronchiectasis	1.487 (0.859–2.575)	0.156		
Presence of COPD	1.681 (1.015–2.784)	0.044		
History of pulmonary TB	1.419 (0.936–2.151)	0.099		
Hemoptysis	0.922 (0.752–1.129)	0.432		
Acid-fast bacilli smear positivity	1.714 (1.161–2.532)	0.007		
*Mycobacterium avium* complex	1.014 (0.637–1.613)	0.955		
*Mycobacterium abscessus* complex	1.348 (0.852–2.133)	0.202		
The single-isolation group	0.316 (0.146–0.681)	0.003	0.400 (0.184–0.871)	0.021
Fibrocavitary type	3.119 (2.003–4.858)	<0.001	2.318 (1.470–3.655)	<0.001

HR, Hazard ratio; CI, confidence interval; BMI, body mass index; COPD, chronic obstructive pulmonary disease; TB, tuberculosis.

## Data Availability

The datasets generated and analyzed for this study are not publicly available but are available from the corresponding author upon reasonable request.

## Supplementary Materials

The following supporting information can be downloaded at: https://www.mdpi.com/article/10.3390/jcm15010296/s1, Table S1: Nontuberculous mycobacteria species concurrently isolated in all patients and newly isolated species during follow-up exceeding one year.
