# Supplementary material for: Concordance Analysis Between Sputum and Bronchoscopic Specimens on Nontuberculous Mycobacteria Pulmonary Disease
Source: J Clin Med. 2025 Dec 30;15(1):296. doi: 10.3390/jcm15010296 (PMC12786755; doi:10.3390/jcm15010296)
Supplement: Supplementary file 1 [file jcm-15-00296-s001.zip › jcm-3971289-supplementary.pdf]

Supplementary Table S1. Nontuberculous mycobacteria species concurrently isolated in all patients and newly isolated species during follow-up exceeding one year

Concurrently isolated species	n = 33
Mycobacterium avium & *Mycobacterium intracellulare*	20 (60.6)
MAC & MABC	8 (24.2)
MAC & *Mycobacterium kansasii*	2 (6.1)
MAC & Miscellaneous	2 (6.1)
Miscellaneous	1 (3.0)
Original and newly isolated species after more than one year	n = 36
MAC & MABC	15 (41.7)
MAC	11 (30.6)
MAC & *Mycobacterium kansasii*	2 (5.6)
MABC & *Mycobacterium kansasii*	1 (2.8)
MABC & *Mycobacterium fortuitum*	1 (2.8)
Three species	6 (16.7)

MAC, *Mycobacterium avium* complex; MABC, *Mycobacterium abscessus* complex
